# Impact of a financial incentive on the completion of educational metrics

**DOI:** 10.1186/s12245-020-00323-8

**Published:** 2020-12-01

**Authors:** Andrew Pugh, Tabitha Ford, Troy Madsen, Christine Carlson, Gerard Doyle, Robert Stephen, Susan Stroud, Megan Fix

**Affiliations:** Division of Emergency Medicine, University of Utah, Salt Lake City, UT USA

## Abstract

**Background:**

The Accreditation Council for Graduate Medical Education (ACGME) requires all emergency medicine (EM) training programs to evaluate resident performance and also requires core faculty to attend didactic conference. Assuring faculty participation in these activities can be challenging. Previously, our institution did not have a formal tracking program nor financial incentive for participation in these activities. In 2017, we initiated an educational dashboard which tracked and published all full-time university faculty conference attendance and participation in resident evaluations and other educational activities.

**Objectives:**

We sought to determine if the implementation of a financially-incentivized educational dashboard would lead to an increase in faculty conference attendance and the number of completed resident evaluations.

**Methods:**

We conducted a pre- and post-intervention observational study at our EM residency training program between July 2017 and July 2019. Participants were 17 full-time EM attendings at one training site. We compared the number of completed online resident evaluations (MedHub) and number of conference days attended (call-in verification) before and after the introduction of our financial incentive in June 2018. The incentive required 100% completion of resident evaluations and at least 25% attendance at eligible didactic conference days. We calculated pre- and post-intervention averages, and comparisons were made using a chi-square test.

**Results:**

Prior to implementation of the intervention, the 90-day resident evaluation completion rate was 71.8%. This increased to 100% after implementation (*p* < 0.001). Conference attendance prior to implementation was 43.8%, which remained unchanged at 41.3% after implementation of the financial incentive (*p* = 0.920).

**Conclusions:**

Attaching a financial incentive to a tracked educational dashboard increased faculty participation in resident evaluations but did not change conference attendance. This difference likely reflects the minimum thresholds required to obtain the financial incentive.

## Introduction

The formative evaluation of emergency medicine (EM) residents by core faculty is a critical component of their professional development [1–3]. The purpose of this process is to assist the resident in recognizing learning gaps and is successful when it inspires residents to improve proactively, developing lifelong learning skills along the way [4–6]. Furthermore, faculty conference attendance is an important part of ongoing development for both residents and faculty, encouraging career-long continuing medical education (CME), fostering relationships between faculty and residents through shared learning experience, as well as providing expert commentary and context to that which is being taught [7, 8]. Participation in these tasks is required by the Accreditation Council for Graduate Medical Education (ACGME), but assuring faculty compliance is challenging [4]. Within our institution and based on annual program review, in 2017, we identified faculty conference attendance and faculty completion of resident evaluations as key areas requiring improvement.

Performance-based financial incentives are widely used in industry and have demonstrated effectiveness as motivational tools, boosting productivity, and attracting and retaining top talent [9, 10]. Performance-based financial incentives are now prevalent in clinical healthcare settings where they have been associated with improved processes of care [11–13]. In academic medicine, we have seen the advent of relative value units (RVUs) for educational activities, with the teaching value unit (TVU) and educational value unit (EVU), but their reported use is currently limited [14, 15].

In June 2018, we introduced a financial incentive for the achievement of certain metrics on an educational dashboard, seeking to determine if the implementation of this intervention would lead to increased evaluation completion rates and faculty conference attendance.

## Methods

### Study design and setting

This study is performed at an urban, academic tertiary facility. The residency is a 3-year program which began in 2005 and consists of 27 residents.

We conducted a 24-month prospective, observational study at our EM residency training program between July 2017 and July 2019. Participants were 17 full-time EM attendings at this single site. This study was IRB approved.

Online resident evaluations were completed on a monthly basis on MedHub, a web-based residency management system designed to track a variety of resident activities, capable of producing reports to monitor compliance with requirements. Conference attendance is facilitated through a system called Check In Help, which requires faculty to call an auto-attendant with a specific event code, recording attendance in real-time. Methods of data collection were the same during the pre- and post-phases of the study. Attendance reports are then provided by the Graduate Medical Education office.

### Dashboard and financial incentive

The educational dashboard is a spreadsheet including metrics such as resident evaluation completion and faculty conference attendance, among others. Data are extracted and compiled from MedHub and Check In Help, displayed in an Microsoft Excel table, converted to final PDF and disseminated to core faculty every three months, a process conducted by the Director of Education and the Residency Manager for the Division of Emergency Medicine. In July 2017, we began publishing the dashboard among core faculty, but at this stage, there was no financial bonus associated. In June 2018, a financial incentive of $1125 per quarter was introduced, thus incentivizing $4500 per annum, approximately 2.5% of annual attending salary. After budgeting, this bonus is deemed to be sustainable long term, beyond the 2-year study period.

Minimum thresholds were established by faculty leadership prior to educational dashboard implementation. In order to receive the quarterly incentive, faculty had to complete 100% of resident evaluations within 90 days and achieve at least 25% attendance at eligible didactic conference days. Conference days are deemed eligible if not scheduled for other conflicting clinical or educational activities (for example, a clinical shift). The average number of evaluations sent to faculty members was consistent throughout the duration of the study at approximately 4 to 6 per month.

### Key outcome measures

We compared the number of completed resident evaluations and conference days attended before and after introduction of our financial incentive. Monthly compliance was collected prospectively over the 24-month period.

### Data analysis

We evaluated the primary outcomes of resident evaluation completion and conference attendance before and after introduction of the incentive using Chi square statistics (vassarstats.net). We additionally report individual faculty compliance rates with conference attendance as a percent of all possible conferences they could attend, taking into account conference days they were working clinically.

## Results

**Fig. 1:**
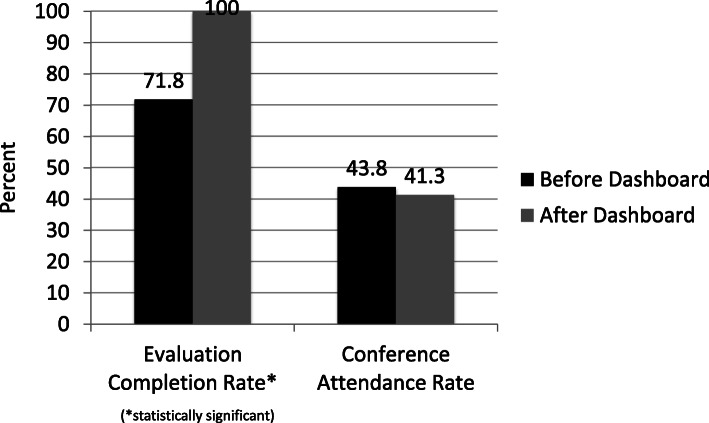
Evaluation completion and conference attendance rates

Over the 24-month study period, we noted significant improvement in the rate of resident evaluation completion. Prior to the incentive implementation, the overall 90-day resident evaluation completion rate was 71.8%. This increased to 100% after the incentive (*p* < 0.001) (Fig. 1). A total of 41.2% (7/17) of our full-time faculty had at least one non-compliant (> 90days) evaluation completion prior to implementation, whereas 0% (0/17) violated the 90-day standard post-implementation. Average time to completion was significantly faster post-dashboard implementation (median 25 days [range 0–90] vs median 51 days [ range 0–360], *p* < 0.001).

Mean conference attendance prior to implementation was 43.8% and did not significantly change 41.3% (*p* = 0.920) (Fig. 1). Of the 17 faculty members studied, 94.1% were compliant with the minimum threshold for conference attendance prior to implementation, unchanged at 94.1% after implementation (16/17). In individuals already compliant, the lowest conference attendance rate was 26.3% prior to the incentive and was unchanged at 26.3% after the incentive. Similarly, among compliant faculty, the highest pre-incentive attendance rate was 68.4% and was 78.3% afterwards. In the faculty member with non-compliant attendance prior to implementation, attendance did not significantly change (13.1% to 14.7%, *p* = 0.920).

## Discussion

We demonstrate that using a financial incentive may be effective in improving specific educational metrics such as evaluation completion rates by faculty. In this case, evaluation completion by faculty improved from 71.8 to 100% after incentive introduction, with median time to evaluation completion improving significantly overall. Although we did not find a change in overall conference attendance, this is likely explained by the fact that 16 of the 17 faculty members studied were already achieving the minimum threshold prior to intervention.

The concept of the financial incentive is not novel, but is being utilized in increasingly innovative and varied settings [16]. In the wake of increased national attention surrounding adverse event reporting, Scott and colleagues sought to investigate the effect of a health and safety initiative which included a financial incentive based on the rate of resident adverse event reporting, with a performance-based resident retirement benefit (1.5% of residents’ annual salaries) [17]. In this prospective cohort study, the approach was successful, as the average number of adverse events reported by residents increased from 1.6 to 9%. Similarly, Conners et al. studied the effectiveness of a financial incentive on patient care, utilizing a pay-for-performance scheme which rewarded pediatric emergency department (ED) providers using a clinical order-set in children with moderate asthma exacerbations [18]. This quality improvement bundle was associated with significantly increased order set use. Further studies have demonstrated that clinical financial incentives can be used to manipulate ED length of stay, documentation efficiency, and other care processes [11, 12, 16, 19].

The use of incentive compensation in academic medicine, in particular academic EM, is less well understood. Changes in healthcare and increasing ED volumes present significant challenges as academic EDs attempt to balance clinical care, research, and education. A systematic review concluded that incentive compensation was associated with positive financial impact and increased productivity in clinical and scholarly activity [16]. In 2015, House and colleagues conducted a prospective observational study to determine the feasibility of an EVU-based system in an academic ED, in which they utilized a taskforce representing educational, research, and clinical missions to identify departmental priorities prior to implementation [15]. In their EVU system, academic activities were assigned standardized time values, with a 30-h threshold to meet the financial incentive. Overall, the authors did notice a significant increase in total EVUs, primarily due to increased conference attendance and evaluation completion at the expense of productivity in other domains, concluding that application of a formal value system to education is feasible. Their work is supported by that of LeMaire et al., who demonstrated similar results using a self-reported academic RVU (aRVU) system among academic surgical faculty [14].

However, there are some important observations from these studies that must be considered. Interestingly, in the House et al. study, individual faculty members who tended not to complete resident evaluations did not change with the new system, implying that some physicians may be less responsive to incentivization than others. This is consistent with our findings, as the one faculty member with low conference attendance (< 25%) did not change after intervention. Furthermore, although it seems intuitive that compensation is a significant predictor of job satisfaction, there is evidence that incentive compensation in academic medicine can harm autonomous motivation and reduce job satisfaction and retention of individual faculty [19–21]. Furthermore, research on clinical incentives has shown that incentivizing performance improvement in one area can lead to deleterious consequences in non-incentivized areas [15, 22, 23]. Systems make trade-offs when selectively incentivizing scholarly activities, and one must weigh the net effect prior to implementation.

In summary, our study and other literature suggests that financial incentives within academic emergency medicine can boost productivity and increase faculty completion of departmental metrics. However, literature suggests the effect of incentivizing tasks needs to be balanced with the risk of reduced compliance with other responsibilities, as well as the effect incentivization may have on morale.

## Limitations

Our study is limited to a relatively small group of EM faculty at a single location, and similar results may not be seen at other institutions with varying faculty composition and compensation structures. Additionally, our study does not address the effect of financially incentivized educational activities on motivation, morale, and job satisfaction, nor does it explore if this detrimentally impacts other professional activities. This is an important consideration, as faculty motivation and morale are critical in ensuring a rich learning environment for residents. Furthermore, our study does not explore how the quality of resident evaluations was impacted; it is conceivable that quantity was attained at the expense of quality evaluation, thus negating the positive impact of increased evaluation completion. Finally, although assumed, we have not directly explored amongst our own residents whether improving conference attendance and evaluation completion improved training experience. This illustrates a need for further research.

## Conclusions

Our study demonstrates that, among a single group of physicians at an academic institution, a financial incentive increased faculty completion rates for resident evaluations. Faculty response to a financial incentive will likely vary based on the minimum threshold required to achieve the incentive.

## Data Availability

The datasets used and/or analyzed during the current study are available from the corresponding author on reasonable request.

## Abbreviations

ACGME: Accreditation Council for Graduate Medical Education
EM: Emergency medicine
CME: Continuing medical education
RVUs: Relative value units
TVU: Teaching value unit
EVU: Educational value unit
ED: Emergency department
aRVU: Academic relative value unit
